# Hidden in plain sound: the scientific potential of house mouse squeaks

**DOI:** 10.1098/rsbl.2025.0333

**Published:** 2025-08-27

**Authors:** Sarah M. Keesom, Lauren R. Leuner, Kayleigh E. Hood, Laura M. Hurley

**Affiliations:** ^1^ Department of Biology, Utica University, Utica, NY, USA; ^2^ Department of Biology, Indiana University Bloomington, Bloomington, IN, USA

**Keywords:** squeak, ultrasonic vocalization, vocal communication, *Mus musculus*, autism spectrum disorder

## Abstract

The house mouse (*Mus musculus*) is an emerging model organism for the study of vocal communication. While mice emit a diversity of calls, most publications on adult mouse vocalizations primarily focus on ultrasonic vocalizations and only a small proportion include other vocalizations, like squeaks. The representation of squeaks in the literature is not an accurate reflection of their behavioural prevalence, however. Squeaks are common features of the mouse vocal repertoire, emitted under a range of circumstances. In this review, we synthesize the available evidence on mouse squeaks, demonstrating that squeaks are social vocalizations. Although their presence in social situations is evident, the extent to which squeaks convey information about the vocalizer and affect listener behaviour across different social contexts has yet to be thoroughly studied. Exploring the nuanced social functions of squeaks and correcting the publication bias that favours ultrasonic vocalizations will require a coordinated research effort, and we provide several recommendations for meeting these goals. Finally, we highlight the potential of the mouse squeak as an instrument for research beyond ethology, including to investigate the neural basis of vocal communication and conditions that impact communication in humans.

## 1. Introduction

**Figure 1.**
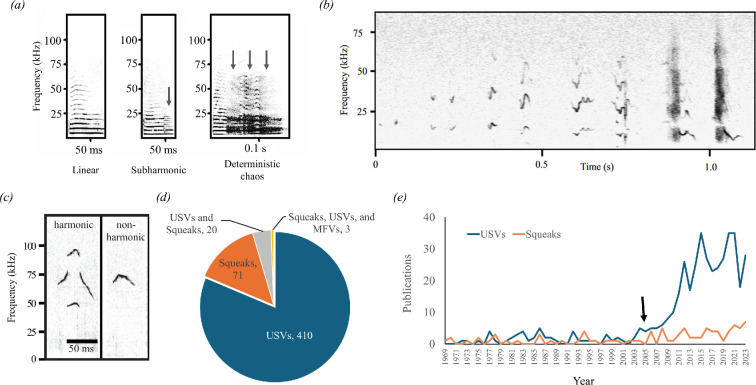
(*a*) Spectrograms of squeaks depicting the presence or absence of nonlinear phenomena: subharmonics and deterministic chaos. Arrows indicate nonlinearities (adapted from [10]). (*b*) Spectrogram of mid-frequency vocalizations [11]. (*c*) Spectrograms of ultrasonic vocalizations (USVs) showing harmonic and non-harmonic USVs (adapted from [12] and used with permission). (*d*) The relative prevalence of publications on the different adult house mouse vocalizations are depicted in panels (*a*–*c*). (*e*) The number of publications (per year) that include USVs (blue line) and squeaks (orange) over time, illustrating an increase in USV-focused publications after 2005 (arrow).

House mice (*Mus musculus*) are among the most widely distributed mammals on the planet, thriving in a range of natural and laboratory settings [1–3]. Consequently, mice are familiar to many biologists, and studies of mice have made substantial contributions to a number of disciplines [1,2,4,5]. One such contribution is their use as a model for studying vocal communication [6–9]. Indeed, mice emit several types of vocalizations, including broadband squeaks ([10]; figure 1*a*), mid-frequency vocalizations (MFVs) ([11]; figure 1*b*), and high-frequency ‘ultrasonic’ vocalizations (USVs) ([6]; figure 1*c*). While MFVs are a relatively recent discovery [11], squeaks and USVs have been studied for decades (figure 1*d*). Following comparisons of USVs to birdsong [13], however, publications focused on USVs have outnumbered those that include squeaks (figure 1*d*–*e*; Box 1). This developing insight into the relevance of USVs to mouse social interactions led to the emergence of USVs as tools to investigate mechanisms of vocal communication (e.g. in mouse models of human conditions that impact communication, such as autism spectrum disorder (ASD) [6,9,33,34]). In contrast, despite their status as arguably the most conspicuous mouse vocalization, a conceptual framework for the social functions of squeaks, and their potential in biological research, is lacking.

Box 1. **Mouse vocalization publication analysis**

A literature search was conducted using PubMed for publications containing the phrase ‘mouse vocalization’, and the resulting publication titles and abstracts were exported. This search was conducted on 15 April 2024 and yielded 1527 papers. Of these, the abstracts and titles were searched to identify if the papers were related to *in vivo* studies of *Mus musculus* (1111 papers) and whether mouse vocalizations were directly studied (829 papers). The resulting 829 publications identified to study *Mus musculus* vocalizations were then categorized by the type of vocalization measured. Because the current review is focused on adult mouse vocalizations, we excluded studies that only investigated USVs made by mice prior to weaning (postnatal day 21). After omitting those papers, we were left with 504 total adult *Mus musculus* vocalization papers published between 1969 and 2024. Of these 504 adult mouse vocalization papers, 410 studied only USVs, 71 focused solely on squeaks, 20 investigated both USVs and squeaks and three other papers reported on a combination of squeaks, USVs, and MFVs (figure 1*d*). Thus, historically, the vast majority of research on adult mouse vocalization has focused exclusively on USVs (81.3% (410/504)), while relatively fewer studies have included squeaks (18.7% (94/504)).

Our literature search for studies investigating *Mus musculus* vocalizations included both wild and laboratory mice. Wild house mice comprise multiple subspecies of *Mus musculus* and have a nearly global presence [2,4,14]. They can thrive at high densities when commensal with humans, as well as low densities when apart from human dwellings [1–3,14,15]. Some experiments use wild-derived mice, referring to descendants of wild-caught mice bred in the laboratory [16–19]. On the other hand, laboratory mice include various strains of mice selectively bred for particular characteristics, as well as genetically modified mice that model human disorders [4,20–24]. With these different populations of mice in mind, it is important to note that behaviours can vary among laboratory strains [25–29], between laboratory and wild mice [17,30–32] and even among populations of wild mice that differ in density or geographic location [1–3]. We therefore evaluated the origin of *Mus musculus* studied in the 504 adult mouse vocalization papers, and we were able to identify the origin of *Mus musculus* in 501 papers. Of these, 488 papers investigated laboratory mice only, 12 papers reported on wild-caught or wild-derived *Mus musculus* and one paper studied both laboratory- and wild-caught mice. Thus, the majority of research on *Mus musculus* vocalizations has been conducted using only laboratory strains of mice (97.6% (488/501)), while fewer studies have used wild-caught or wild-derived mice (2.6% (13/501)).

In this review, we present the mouse squeak as a social behaviour with considerable research promise. We begin by comparing the acoustic structures and production mechanisms of different mouse vocalizations, then provide a synthesis regarding the functions of squeaks across behavioural contexts. We consider possible explanations for the unequal representation of squeaks and USVs in the literature, and we offer recommendations for improving the study of the entire mouse vocal repertoire. Finally, we conclude by describing how translational studies that employ mice as a model organism would benefit from the inclusion of squeaks, the mouse vocalizations that are ‘hidden in plain sound’.

## 2. Adult house mouse vocalizations

In this paper, we consider vocalizations emitted by adult house mice: squeaks, MFVs and USVs. USVs occur exclusively at high frequencies (>20 kHz) with a narrowband structure [6–8,20,21,25,35–38], although harmonics, vocal noise and sidebands are sometimes observed [7,13,16,21,35–37,39–45]. In contrast to USVs, numerous terms have been employed to refer to the broadband vocalizations made by mice (table 1). We refer to this call type as a ‘squeak’, because this term describes the sound, instead of ascribing a function to the call or referencing its detectability by humans. Squeaks possess a fundamental frequency between 3 and 4 kHz and a dense harmonic structure extending into higher frequencies [10,46,58], rendering the acoustic structure of squeaks strikingly distinct from that of USVs. As a separate category, MFVs are defined by a fundamental frequency intermediate to squeaks and USVs [11,16,39]. Because MFVs have only recently been identified and have thus received less attention, we primarily compare squeaks and USVs.

**Table 1.** Terminology used in the literature in reference to the mouse vocalizations that we call ‘squeaks’ in this paper.

basis of terminology	term	references
detectability by humans	audible call	[46]
	audible cry	[47]
	audible vocalization	[6,26,48,49]
	sonic vocalization	[27,50,51]
assumed function	defensive call	[46]
	distress vocalization/call	[26,52]
	fright call	[53]
	rejection call	[54]
	stress call	[55]
	submissive vocalization	[53,56]
spectral structure	harmonic call	[57]
	low-frequency harmonic	[11,39,58]
	broadband vocalization	[10,16,59–61]
onomatopoeia	click	[25]
	squeak	[12,27,56,62–71]
	squeal	[57,71–79]

The structural distinctions between squeaks and USVs arise from different modes of production in the vocal tract. USVs are generated through a whistle-like mechanism arising from a compressed airstream that is modified by interaction with vocal specializations [80–83]. Unlike USVs, squeaks are produced in the same way as many mammalian vocalizations, from the calls of small rodents and primates to the infrasound of elephants to human speech: through the vibration of vocal folds [54,84–87]. Both processes can give rise to a remarkable range of variation in acoustic structure including modulations in frequency, amplitude and duration; each process can additionally generate nonlinearities like frequency jumps or abrupt changes in harmonic structure [13,35,46,88]. For squeaks, one of the most well-characterized types of acoustic variation is related to irregular or out-of-phase vocal fold vibrations that may appear as an abrupt doubling of harmonics (subharmonics) or structured noise (deterministic chaos) on spectrograms (figure 1*a*) [10,46,89,90].

Structural variation among squeaks and USVs has been categorized in fundamentally different ways. USVs are often placed in discrete categories based on statistically significant clustering in frequency modulation, frequency jumps and the presence or absence of harmonics; however, other analyses suggest a gradation of call structures among classes [13,21,35,88,91–94]. Certain categories of USVs show correspondence to context and behavioural distinguishability [16,35,36]. In contrast, variation among squeaks is commonly quantified as differences in qualities like duration and frequency. A few authors have compared characteristics of squeaks across different contexts [10,58,72], but distinct categories of squeaks remain unresolved. For example, several papers have distinguished between squeaks with and without nonlinearities [10,16,46], but it is not clear whether variation in nonlinear phenomena among squeaks is continuous or categorical. Furthermore, whether squeak structural variation corresponds to behavioural responses to squeaks is almost entirely unexplored.

## 3. Functional hypotheses for squeaks across contexts

Squeaks are ubiquitous mouse vocalizations, occurring in a variety of situations. While squeaks are also associated with general distress and can be elicited by scientists through the application of aversive stimuli [26,27,53,58,72,95,96], our focus is on the significance of squeaks to ethologically relevant contexts. We therefore draw on studies of both wild-caught *Mus musculus* and laboratory strains of mice, although most studies of *Mus musculus* vocalizations use laboratory mice (Box 1). Thus, in this section, we present hypotheses regarding the social functions of squeaks during courtship, same-sex social encounters and predator confrontation, and we highlight findings from wild or wild-derived *Mus musculus* where possible.

### (a) Courtship interactions

Some of the first hints that vocalizations are involved in mouse courtship were obtained from observing how vocalizations correspond to non-vocal behaviours [47,62,73]. While males are the primary emitters of USVs when the two sexes interact, female mice have recently been discovered to contribute a small proportion of USVs [97–99]. Female vocalizations during courtship are therefore beginning to be acknowledged, but USVs are only one aspect of the female vocal repertoire. Squeaks are common elements of mouse courtship-related behaviour and are uttered in a majority of mixed-sex interactions [10,12,52,100], primarily by female mice [63]. Despite the prevalence of squeaks, their function remains ambiguous, although the limited data from both laboratory and wild-derived mice suggest that squeaks play an important role in encounters between the sexes.

**Figure 2.**
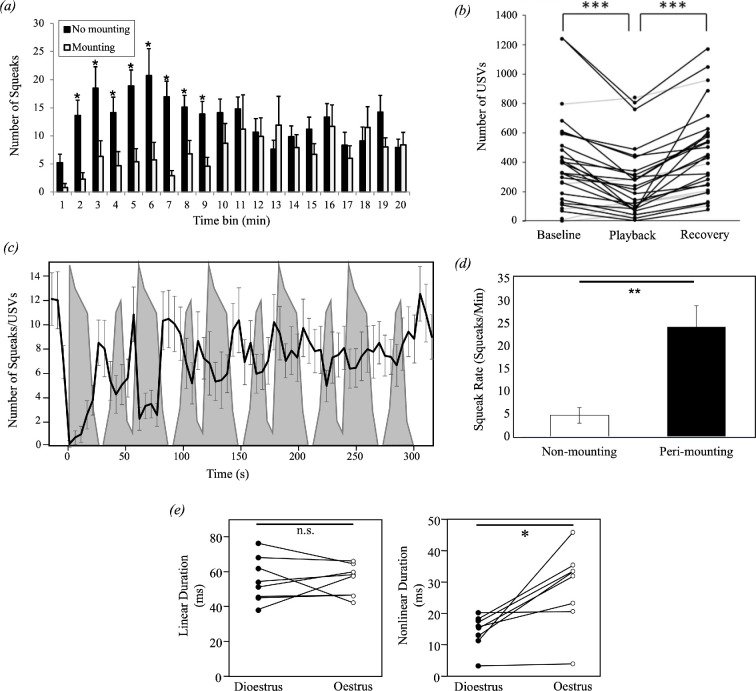
(*a*) Female squeak production is higher at the beginning of mixed-sex interactions in which mounting did not occur (black bars) compared to interactions in which mounting did occur (open bars) (adapted from [10]). (*b*) USV emission by male mice is significantly lower during female squeak playback compared to the preceding baseline period with no playback [59]. (*c*) USV emission by male mice (black line) is quickly suppressed by female squeak playback (grey shading), but rapidly recovers during silent periods (adapted from [59]). (*d*) Female squeaking rate is significantly greater in time-bins surrounding mounting incidences (peri-mounting) compared to non-mounting time-bins (adapted from [10]). (*e*) Female squeak structure changes across the oestrous cycle. Unlike linear portions of squeaks (left panel), nonlinear segment duration significantly increases during oestrus compared to dioestrus (right panel) [10].

Several observations indicate that female squeaks during courtship may be signals that convey negative affect. Anecdotal reports note a temporal correspondence between female squeaks and male aggression [101] or with female defensive behaviours [10,46,48,63,73,74,101]. Likewise, there is a significant positive correlation between the total number of male-repelling behaviours and the total number of female squeaks recorded from an interaction [10,12,25]. Female mice also make more squeaks towards males infected with bacteria compared to healthy males [101], and a high rate of squeaking by a female mouse at interaction onset is associated with a lower likelihood of male reproductive behaviours at later time points ([10]; figure 2*a*). Similarly, in wild-derived mice, squeak rate is higher in the second before a failed mounting attempt compared to successful copulation [16]. Consistent with the interpretation of squeaks as negatively valenced signals during courtship, the number of vocalizing males decreases during squeak playback when female odours are absent [64]. In the presence of female odours, playback of squeaks suppresses male USVs ([59]; figure 2*b*), and this reduction in USVs is driven by an immediate decrease in vocalization during portions of the audio file that contains squeaks, emphasizing that male mice rapidly respond to hearing squeaks (figure 2*c*). In keeping with these observations, authors have often interpreted female squeaks emitted during courtship as signals of negative valence, indicative of decreased sexual receptivity or rejection of the male [12,25,54,62,63].

Squeaks emitted by females during courtship may have multiple functions, however, given that female squeaks can also be positively related to male behaviours that increase the likelihood of mating [10,16,63]. For instance, male mice approach squeaks when squeak playback is paired with female mouse urine, despite avoiding squeaks in the presence of predator odours [58]. During male–female interactions in which mounting occurs, the female squeaking rate increases around mounting events in the latter part of the encounter ([10]; figure 2*d*), and a similar pattern has been described in wild-derived mice [16]. Additionally, in courtship encounters of both laboratory and wild-derived mice, squeaks can be found alternating or overlapping with USVs [10,16,46,52,63], especially around a mounting event [10,63]. In other words, female squeaks are correlated with male behaviour differently, depending on the valence of the encounter and the time point within that interaction. Squeaks emitted when a female first contacts a male are negatively associated with mounting later on; thus, a high number of squeaks at interaction onset may convey rejection. Considering that female mice regulate the rate of copulation with a male when possible [48] and that males rapidly suppress their vocalizations in response to female squeaks [59,60], a speculative function of squeaks emitted at later time points is that they could serve to provide feedback to males [102] or slow the advances of the male mouse [103], especially when the female is unable to escape.

The idea that female squeaks may have multiple functions within a courtship context is further supported by observations that squeaks exhibit context-dependent variation in spectrotemporal structure for both wild-derived [16] and laboratory mice [10]. Nonlinear squeaks increase at later time points during courtship [16], and around mounting incidents, squeaks contain longer-duration nonlinear segments compared to squeaks emitted at other times [10]. Additional variability in nonlinear phenomena within squeaks is evident across the oestrous cycle [10]. Female mice produce squeaks with longer nonlinear elements when they are in oestrus, and thus, sexually receptive, compared to when they are in dioestrus ([10]; figure 2*e*). Taken together, these results suggest that nonlinear phenomena may convey increased female receptivity, given that squeaks with increased nonlinearities are more likely to emanate from female mice that are physiologically receptive (i.e. in oestrus) and behaviourally receptive (i.e. allowing mounting). An important unanswered question, then, is whether male mice respond differently to squeaks based on their nonlinear content.

### (b) Same-sex social interactions

In addition to courtship, mice readily vocalize upon encountering a mouse of the same sex. While both sexes make USVs when engaging with a same-sex conspecific [57,104], males are more likely to squeak during same-sex interactions [57,65,72]. In this context, squeaks likely originate from the recipient of aggression [53,57,66,67,72,75,105], showing a strong correspondence with incidences of agonistic behaviour [56,106]. Mice squeak when bitten [105], tussling with another mouse [72] and assuming a defensive posture when confronted with an aggressive mouse [57,76]. Younger mice are more likely to employ squeaks [67], and early-life defeat enhances squeaking in adults [65,75]. Size of the arena influences vocalization, with mice less likely to squeak in a larger arena when studied in laboratory settings [67]. Same-sex encounters staged in a laboratory setting also elicit squeaks from wild-caught mice [68,76], and the use of squeaks in this context may vary depending on whether mice were living ferally or commensally with humans when captured [68]. Thus, in both laboratory and wild-caught mice, squeak emission during same-sex encounters may be context-dependent; however, additional research is required to further validate this idea.

A limited number of studies suggest that squeaks influence the behaviour of same-sex recipients. Following squeak emission by subordinate mice, aggressive mice have occasionally been observed to withdraw without attacking, suggesting that squeaks are not only elicited by painful stimuli but may function to prevent attacks [67]. Squeaks are not always aversive in a same-sex context, however. While male mice are repelled by squeak playback in the absence of another mouse, they are attracted to squeaks when a male stimulus is present [27]. Thus, similar to courtship, squeaks may be multifunctional within a same-sex context, and the receiver’s response to squeaks may depend on non-auditory contextual cues. It is additionally possible that the acoustic properties of squeaks could affect receiver behaviour. For example, when two male mice interact, they emit squeaks with a range of nonlinearities [90]; however, whether nonlinearities affect male receiver behavior has yet to be tested.

### (c) Predator confrontation

Beyond conspecific interactions, mice squeak when confronted with a predator, such as a rat [30,50,107–109]. The likelihood of predator-evoked vocalization varies among laboratory strains, as well as between laboratory mice and mice derived from wild populations when recorded in the laboratory [30,107]. When provided ample space, wild-derived mice vocalize less and spend more time fleeing a predator compared to laboratory mice; however, when unable to escape, wild-derived mice vocalize more than laboratory strains [30]. Thus, predator-elicited squeaks may depend not only on the mouse’s background, but also on the environment and ability of the mouse to employ other anti-predator tactics.

In this context, it is possible that squeaks may serve as alarm signals to conspecifics. In the absence of olfactory cues, hearing squeaks triggers elevated corticosterone, a hormone involved in the coordinated stress response [39]. Mice also exhibit increased heart rates while listening to the squeaks of conspecifics experiencing an aversive stimulus [26]. At the level of behaviour, squeak playback in the absence of conspecific chemical cues or presence of predator cues increases avoidance behaviour in a Y-maze [27,58] and in an open field [39]. Mice develop a behavioural fear response to a neutral stimulus when it is paired with squeak playback [26], suggesting that hearing the squeaks of another mouse encountering a predator could facilitate learned avoidance of predator cues. Because mice squeak in response to predators [30,50,107–109], and predators are attracted to squeaks [27], squeaks could indicate that a predator is present at that location. Taken together, these studies suggest that, depending on contextual cues, squeaks can activate physiological processes and behaviors that could increase survival of the listener.

### (d) Summary: functional hypotheses for squeaks

**Figure 3.**
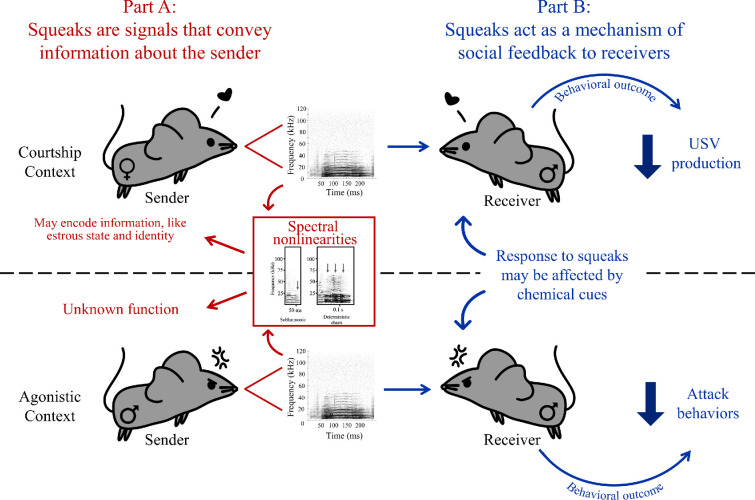
Conceptual diagram depicting functional hypotheses for mouse squeaks.

Mouse squeaks are social signals, and the existing evidence further hints that squeaks may have multiple functions across social contexts. We therefore suggest several functional hypotheses in which squeaks should convey information about the vocalizer and influence the behaviour of listening animals in different situations (figure 3; Box 2). Recalling that publications on squeaks are fewer than those that focus on USVs, these hypotheses suggest crucial areas for future studies that describe how variation in squeak acoustic structure relates to qualities of the sender, as well as research that investigates how receivers respond to squeaks with varying spectrotemporal characteristics in diverse behavioural contexts. It is critical to note that few studies have measured vocalizations emitted by wild-caught or wild-derived mice, so comparisons between wild and laboratory mouse squeaks must be made with caution. Less is known regarding vocalizations produced by house mice in free-living populations (but see [110]). Future research should therefore investigate the extent to which the hypothesized functions of squeaks extend to wild *Mus musculus* outside the laboratory.

Box 2. **Functional hypotheses**

The current evidence demonstrates that mouse squeaks are social vocalizations, yet the nuanced roles of squeaks in mouse vocal exchanges remain to be fully explored. Here, we present functional hypotheses for squeaks across contexts: that squeaks communicate information about the vocalizer (Part A) and influence the behaviour of the listening animal (Part B).

Part A: Squeaks are communicative signals that convey information about the sender, including identity, physiological state and affect. This includes conspecific interactions and encounters with predators. Nonlinear phenomena may be particularly salient features of squeaks. During courtship, nonlinearities in female squeaks could function to communicate the identity and estrous state of female mice [10]. During male–male agonistic encounters, squeaks also contain nonlinear elements [90], but no data exist regarding their potential function in this context or during predator confrontation. Thus, one intriguing future avenue of research on potential functions of spectrotemporal variation in squeaks would be to carefully document the presence of nonlinearities and their association with non-vocal behaviours in contexts other than courtship.

Part B: Squeaks act as a real-time mechanism of social feedback to receivers across different contexts. During male–female courtship encounters, squeaks produced by females rapidly alter the behaviour of the male, including the production of USVs [39,59,60], which may be one behaviour used by females to regulate the timing of copulation. In male–male agonistic encounters, squeaks emitted by males may halt or reduce attacks [67]; however, playback studies are needed for this context. Finally, given that male mice respond differently to squeak playback depending on chemical and visual cues [27,58,64], non-auditory contextual information may play an important role in allowing mice to respond appropriately to a squeak. The factors that contribute to variation in receiver responses to squeaks can therefore be evaluated by using playback studies that manipulate non-acoustic cues or manipulate different spectrotemporal parameters of squeaks, including nonlinearities.

Future research should test these functional hypotheses with mice in laboratory settings as well as in the wild. While some studies have allowed for comparisons between laboratory and wild-derived mice (e.g. [10,16]), the majority of published research focuses on *Mus musculus* vocalizations recorded in laboratories (Box 1). Thus, one critical goal for the study of mouse vocal signalling will be to validate functions of squeaks determined using different strains of laboratory mice by testing those functions in wild populations of mice under natural conditions. Indeed, a recent study by Jourjine *et al.* [110] demonstrates that, in one population of free-living *Mus musculus,* USVs and squeaks correspond with changes in social grouping; however, additional research is needed to further determine the social functions of vocalizations in wild house mice across different geographic locations and population densities. Establishing the functions of mouse squeaks across contexts will improve our understanding of the social behaviour of mice and, consequently, enhance the translational value of mouse vocal communication.

## 4. Optimizing the potential of squeaks in research

It is notable that the mouse vocalization humans can hear without aid, a squeak, is not the vocalization that is the most well studied. In this section, we provide recommendations for rectifying the research disparity between squeaks and USVs, based on possible sources of the present publication bias.

### (a) Conduct more descriptive studies of squeaks

#### (i) Source of bias

One plausible explanation for the gap between studies of squeaks and USVs may relate to differences in spectrotemporal characteristics between the two call types. Because they contain lower frequencies, squeaks are conspicuous to humans and can be associated with other behaviours without special equipment; thus, squeaks may have been disregarded because their function is assumed. Indeed, numerous functions have been attributed to squeaks (table 1), although most have yet to be rigorously tested with playback studies that manipulate characteristics of squeaks. In contrast, some researchers may be intrinsically curious about USVs due to their inaudibility to humans, which imparts USVs with the quality of a seemingly ‘secret’ mode of communication (see [111] for a similar idea related to avian ultraviolet signals). Furthermore, USVs are strikingly variable in frequency, duration and timing when depicted on a spectrogram and have been compared to birdsong. This comparison, highlighted in 2005 by Holy & Guo [13], was followed by an increase in USV-focused publications (figure 1*e*). Thus, differences in the spectrotemporal parameters of these two call types may have contributed to the neglect of squeaks in research involving mouse vocalizations.

#### (ii) Recommendation

Scientists should draw attention to squeaks through detailed quantification of these calls across contexts, such as courtship, aggression and predator confrontation (e.g. [10,16,58]). The thorough characterization of vocalizations recorded from mice in both laboratory and natural settings will facilitate the development of testable hypotheses regarding the nuanced functions of squeaks, including different spectrotemporal elements like nonlinearities. Uploading descriptive data on squeaks from mice of various backgrounds to large-scale projects like the Mouse Phenome Database [112] could help delineate gene–behaviour relationships for vocal communication.

### (b) Develop tools for automated analysis of mouse vocalizations in noise

#### (i) Source of bias

Manual spectrographic analysis of vocalizations is labour-intensive (e.g. during courtship, male mice can emit 90−210 USVs per minute [113–115]). Advancements in machine learning have therefore facilitated a critical improvement by enabling high-throughput analysis of USVs [21,91–93]. However, the same methods used to automatically quantify USVs may inadvertently exclude lower-frequency vocalizations, such as squeaks and MFVs, if a high-pass filter is used to enhance USV detection (e.g. [98,114,116]). This is problematic for analysis of USVs recorded from social interactions in which squeaks can occur. Playback experiments demonstrate that male mice alter USV emission in response to squeaks [39,59,60], suggesting that squeaks may influence USVs during direct social encounters. Thus, a complete understanding of mouse vocal communication may only be attained if all vocalization types are included in analyses.

#### (ii) Recommendation

When researchers investigate the temporal dynamics of mouse vocal communication during direct social interactions, squeaks and MFVs should be included if they are present. To that end, scientists can adapt available automated tools for vocalization analysis (e.g. [117,118]) to identify squeaks, as has been accomplished by Jourjine and colleagues [110]. We further suggest that researchers take care to reduce possible noise sources when designing experiments and that tools for vocalization analysis be improved to detect mouse calls even in the presence of background noise so that high-pass filtering need not be employed. This will allow researchers to leverage their rich datasets to make detailed observations from which new hypotheses about mouse vocal communication can be derived [119].

### (c) Employ more female-centred study designs for research in courtship context

#### (i) Source of bias

Because mouse vocalizations are most frequently studied during courtship, when males predominantly utter USVs, and females emit squeaks [63], the greater research focus on USVs could in part be following the historical precedent of investigating courtship from a male point-of-view. Male animals have received the most attention in the study of courtship since Charles Darwin proposed the theory of sexual selection [120,121]. A prevailing belief among scientists contemporary with Darwin was that female animals were ‘coy’ and ‘passive’, whereas males were described as actively pursuing females [120–122]. Although progress is being made [103,123,124], this ‘female passive, male active’ framework continues to influence thought surrounding sexual selection and reproductive biology, including courtship behaviours [120,122,123,125]. A failure to consider a female perspective risks the perpetuation of stereotypes based on assumed sex roles and impairs the accurate interpretation of results. For mouse courtship, studying females is increasingly common via analysis of their USVs [97–99], but the full female vocal repertoire, which includes squeaks, is less often considered. It is thus possible that a bias inherent to the study of courtship translated to the study of mouse vocalizations in other contexts.

#### (ii) Recommendation

Given the prevalence of mouse courtship as a paradigm used to study communication, we recommend re-evaluating the role for female mice during reproductive encounters. By comparison, it is now recognized that female song exists in most songbird species, and female birds are active participants in reproduction [123,126]. Consistent with these insights in songbirds, we suggest that researchers studying mouse courtship vocalizations employ more female-centred study designs, such as playback experiments with a Y-choice assay [58] or split-cage assay [59,60] to assess how males respond to female squeaks possessing different acoustic characteristics. Research in this vein will help determine how both sexes contribute to courtship in mice, leading to an increasingly sex-inclusive understanding of communication in this context.

### (d) Carry out experiments to investigate how squeaks affect receivers across contexts

#### (i) Source of bias

The existing literature can influence future research conducted, and this may be true for research on the social functions of mouse vocalizations. A sizable body of literature exists on behavioural responses to USVs and USVs with different properties [17,18,25,64,127–134], including USVs from mice of different laboratory strains [129,134] or separate species [18]. This has conceivably driven a positive feedback cycle in which even more research is conducted with USVs. While a few experiments have evaluated receiver responses to squeaks in general [27,39,58–60], whether mice respond differently to squeaks from other species or with varying acoustic characteristics has yet to be thoroughly investigated. Thus, the limited information on how squeaks affect receivers likely influences the extent to which scientists view squeaks as communicative signals, possibly contributing to the exclusion of squeaks from some studies.

#### (ii) Recommendation

Researchers should conduct experiments to investigate how squeaks, and variation in properties of squeaks, affect receivers in both laboratory strains and mice from wild populations. Operant conditioning assays should be conducted to determine whether mice can discriminate between squeaks of varying spectrotemporal characteristics, as has been accomplished with USVs [131,135–137]. Playback experiments that broadcast squeaks from multiple species or manipulate qualities of squeaks in combination with behavioural assays (e.g. Y-maze, split-cage) will provide crucial insight into the functions of squeaks. While there has been some investigation of squeaks in a courtship context, researchers should also employ playback experiments to ascertain the functions of squeaks during encounters with same-sex conspecifics and with predators (but see [27]).

## 5. Squeaks as a promising tool for neuroscience and biomedical research

The improvement of research conducted on mouse vocalizations will not only facilitate a deeper understanding of the vocalizations themselves but also improve the applicability of mice as a model to study communication. The acoustic and productional similarities of mouse squeaks to the airflow-induced vocal fold vibration sounds of other mammals suggest that squeaks would have high translational value across different areas of communication research. Here, we highlight how squeaks could enhance studies that use mice to investigate the neural basis of vocal communication and human conditions that affect communication.

### (a) Neural mechanisms of vocal communication

Research using USVs to study the neural basis of vocal communication has provided significant insight into how the nervous system controls sound generation and reception. However, a few recent studies provide tantalizing evidence that the neural pathways that regulate squeaks and USVs are parallel to but distinct from one another. A central region for vocal production is the midbrain periaqueductal grey (PAG), which has separate populations of neurons that, when stimulated, can elicit squeaks or USVs [52,138,139]. The role of PAG neurons in vocal production has been described as ‘gating’, because they cause vocalizations when directly stimulated and prevent them when inhibited, with little effect on vocalization structure [140]. PAG neurons regulating squeaks and USVs receive converging inputs from upstream regions including amygdalar, cortical and hypothalamic areas, which confer sensitivity to social or threatening behavioural contexts [139–141]. Downstream of the PAG, there is some degree of functional convergence of squeak- and USV-generating circuitry to regions like the nucleus retroambiguus (RAm), a premotor nucleus that provides input to neurons controlling the larynx and respiratory cycles [138,142]. Silencing RAm neurons that are active during USV production prevents both USVs and squeaks from occurring during relevant contexts, although stimulating these neurons optogenetically only elicits USVs, not squeaks [142]. Overall, these neural circuits create opportunities for the separate regulation of squeaks and USVs that parallel their differences in behavioural function.

The dissimilar acoustic structures of squeaks and USVs also necessitate distinct processing mechanisms for these two calls by the central auditory system. One notable organizational feature of auditory regions from the cochlear nucleus through the auditory cortex is frequency mapping (e.g. [143–145]). This fundamental organizational principle has several consequences for the representation of squeaks versus USVs in auditory brain regions, some of which have been explored in the auditory midbrain. The first difference is that squeaks activate neurons in areas of the frequency map that are distinct from USVs [146]. Because squeaks typically span a broader frequency range than USVs, single neurons are likely to respond to multiple examples of squeaks [61,146], similar to the low selectivity for broadband calls observed in other species [147–149]. On the other hand, auditory neurons in some regions show a unique type of selectivity for squeaks in that they have distinct temporal responses to different squeaks, suggesting that they are selective for specific spectrotemporal features of squeaks that are variable among calls [61]. The structural elements that contain behaviourally relevant information in squeaks might therefore be represented by temporal patterns of activity across broad segments of a population of auditory neurons.

A second aspect of frequency tuning with consequences for squeak responses is the disproportionate number of neurons responding to lower frequencies versus frequencies above 50 kHz, although this proportion decreases from the periphery to the auditory midbrain [150,151]. Interactions among cochlear responses to the high frequencies in USVs could create distortion products at lower frequencies, allowing neurons tuned to low frequencies to respond to USVs [152,153]. However, neural responses in the auditory midbrain are predicted well by the overlap between frequency tuning and the frequencies within a call [146]. As a result, more neurons respond to squeaks, MFVs or USVs with lower-frequency features than to higher-frequency USVs [146]. The frequency map in the auditory regions of mice may therefore translate into a functional map dominated by responses to lower-frequency calls like squeaks.

Taken together, the existing data suggest that studying the neural basis of USV production and reception alone may only reveal specialized neural processes in rodents. Because squeaks show acoustic and peripheral productional similarities with other mammalian vocalizations, the increased inclusion of squeaks could allow neuroscientists to distinguish between conserved and derived mechanisms of vocal communication.

### (b) Human conditions: autism spectrum disorder

Mouse vocalizations have provided tractable models for research on human communication, social disorders and other health concerns [6,154,155]. Squeaks have been used to investigate affective conditions like distress and anxiety [26,27,55,66,96,156,157] and have been used as indicators of pain [95,158–160], as well as seizures [20]. In the applied study of social communication, like ASD, USVs during dyadic male–female interactions are a well-established paradigm [24,88,155,161,162]. ASD is a neurological condition with a primary diagnostic criterion of social communication deficits [22,155,163–166]. Characterization of the USVs emitted by ASD model mice has been important for establishing links between ultrasonic communication deficits in ASD mice and communication irregularities displayed by autistic people [24,88,161,167]. Specifically, studies emphasize how ASD mice respond differently to contextual information and social experiences [33,34,166]. For example, while sexual and social experience significantly increase vocal behaviour in wild-type laboratory mice, ASD model mice still exhibit low rates of USVs after experience with females [166].

Contrary to the focus on USVs, applied studies that assess male mouse vocalizations during mixed-sex interactions seldom account for the female partner’s behaviour [33,34,167–171], and squeaks generally remain unanalysed, despite the likelihood of their occurrence. Given that squeaks have the potential to shift the dynamic and outcome of a courtship interaction [10,16,59,60,64], the inclusion of squeaks in translational research, especially in experiments that employ assays where mice directly interact, may shed new light on the human conditions under investigation. This is particularly relevant to the study of ASD, because murine models of ASD and human subjects with ASD show increased sensitivity to sound, and the type of sound may affect whether the individual has a negative reaction to it [162,172,173]. For example, autistic children and adults display differences in vocal emotional perception compared to non-autistic individuals [174,175]. Because squeaks are sounds that potentially convey female affect, it is possible that ASD model mice may differ from wild-type mice in their responses to squeaks. This could explain differences between mouse models of ASD and wild-type mice in male behaviours observed during direct interactions when unanalysed squeaks might be present [33,34,167–171]. Thus, the behaviour of female mice during engagement with male ASD model mice should be considered, and the strong behavioural salience and prevalence of squeaks during opposite-sex interactions make squeaks a prime candidate for investigating how ASD mice respond to social signals. Since it is possible that male ASD model mice could squeak when approached by females, synchronized microphones and cameras should be employed to determine which individuals produce squeaks during direct interactions. Furthermore, because USVs, MFVs and squeaks likely function differently, evaluating how mouse models of ASD respond to all types of vocalizations, not just USVs, could offer insight into how individuals with ASD process vocal signals of varying meaning.

## 6. Conclusion

There is accumulating evidence that squeaks are important social vocalizations for mice, yet squeaks await the level of attention that has been afforded to USVs. The inclusion of squeaks in basic and applied vocal communication research offers many exciting opportunities, and we therefore encourage scientists to integrate squeaks, as part of the entire mouse vocal repertoire, into their research agendas. Incorporation of squeaks will make results from vocal communication studies using house mice more useful to the scientific community, more translatable and more inclusive of the general population.

## Data Availability

The data from the publication analysis are included as electronic supplementary material. The Microsoft Excel file contains the data, and the metadata file describes the data contained in the Excel file, as well as the analysis performed. Supplementary material is available online [176].
